# One size does not fit all: Preferences for HIV care delivery among out-of-care people living with HIV in the Southeastern United States

**DOI:** 10.1371/journal.pone.0276852

**Published:** 2023-01-17

**Authors:** Marxavian D. Jones, Kelly Dyer, Emma R. Nedell, Michelle R. Fletcher, Cassie Grimsley Ackerley, Sophia A. Hussen, Ameeta S. Kalokhe

**Affiliations:** 1 Hubert Department of Global Health, Emory University Rollins School of Public Health, Atlanta, GA, United States of America; 2 Division of Infectious Diseases, Emory University School of Medicine, Atlanta, GA, United States of America; University of Miami, UNITED STATES

## Abstract

Approximately half of the people with HIV (PWH) in the United States are retained in HIV care and only 57% have achieved viral suppression, due to barriers including transportation access, stigma, poor mental health, substance use, and medical mistrust. Community-based HIV care models have potential to address the diverse needs of patients and to improve retention in care, but their success is contingent on acceptance by patients and key community stakeholders. Recognizing that the preferences of PWH who are out-of-care (PWH-OOC) likely differ from those retained in care, we conducted a mixed-methods study from June 2019 to May 2021 composed of surveys with PWH-OOC (n = 50) and in-depth interviews with key clinic and community stakeholders (n = 41) to examine the relative preference and perceived advantages and disadvantages for six different community-based HIV care models versus the traditional fixed-clinic model. Survey data was analyzed to assess average rank preference for each care model and interview transcripts were thematically coded to examine factors influencing model acceptance. The highest preference for care delivery was via a mobile clinic, followed by community-based peer navigation, primary care clinics, telemedicine, traditional HIV subspeciality clinic, homeless shelter, and drug treatment center. Common factors influencing preference included convenience, accessibility, potential to preserve confidentiality, quality of care assurance, opportunity to develop rapport with their HIV care provider, access to a smart device, and potential to alleviate versus exacerbate HIV stigma. Participants discussed need for integration of care models and for individuals to choose different care models at different times. Providers and patients differed in preference for care model and weighting of relative advantages and disadvantages of each. Findings highlight the need to integrate alternative, community-based care models into the national plan to end the HIV epidemic and to allow for PWH-OOC to choose the model most fitting based on individual circumstances.

## Introduction

Despite ongoing efforts to enhance the HIV care continuum, only half of the people living with HIV (PWH) in the United States (US) are retained in HIV care [1,2]. Retention in care is a critically important stage of the continuum that strongly predicts viral suppression, progression to AIDS, non-AIDS complications, mortality, and HIV transmission [3–7]. As a consequence of low rates of retention and viral suppression, the HIV epidemic continues with roughly 37,000 new diagnoses occurring each year in the United States [8]. The southern United States, plagued by high levels of poverty, racism, transphobia/homophobia, and poor access to healthcare, is home to eight of ten states with the highest rates of new HIV/AIDS diagnoses [9], underscoring the need for new, innovative community-tailored approaches to improve retention in HIV care in the South.

In 2019, the US Department of Health and Human Services established the Ending the HIV Epidemic (EHE) initiative, with an express goal of developing and implementing “innovative, community-driven solutions to leverage scientific advances in HIV prevention, diagnosis, treatment and outbreak response [10].” The EHE initiative further identified 50 local jurisdictions to target for intensive efforts, including four counties in the metropolitan Atlanta area, 20 counties in the South and seven entire southern states. To achieve the EHE goals, novel approaches to enhance retention should comprehensively target known barriers to HIV care, including transportation vulnerability, fear, stigma, poor mental health, physical health limitations, substance abuse, and the complexities of navigating a fragmented health system [11–14]. Ideally, care delivery models should simplify care access by addressing the geographic inaccessibility posed by inadequate public transit in the South [15–19], render HIV care more emotionally safe, comfortable, and pleasant for PWH by addressing stigma and historic mistrust, and build trust with PWH and the community at large. Accordingly, the CDC emphasizes the need to design care strategies to “meet patients where they are” through bringing HIV care to the community [20].

Numerous innovative alternative and community-based HIV care delivery models have been developed to address care engagement and retention worldwide, but have been implemented in the Southern US to varying degrees. For example, in Africa, mobile clinics (wherein care is provided in the community within the privacy of a vehicle) have been used in Mozambique [21], Kenya [22] and Uganda [23] to deliver HIV care to under-served and hard-to-reach populations. In rural Kenya, peers living with HIV deliver antiretroviral therapy to patient homes [24]. Community-based patient navigation (wherein a peer with HIV helps support the patients access HIV care from home) has been associated with enhanced linkage to care, retention and viral suppression [25,26]. Telemedicine (wherein care is provided via a private online platform) has shown promise, albeit with challenges of its own, apparent through widespread use during the COVID-19 pandemic [27,28]. Lastly, comprehensive “one-stop shops” with integrated HIV, non-HIV care, and support services have been shown to increase access to care by fostering convenience when comorbidities require multiple appointments [29,30].

While these innovative care models have significant potential to enhance retention in HIV care, their success is contingent on their acceptance by patients—particularly PWH who are out-of-care (PWH-OOC)—and community members, feasibility of implementation, and safety. Further, the care delivery preferences of PWH-OOC may be very different from those who are well-engaged in HIV care. To date, there have been limited attempts to understand the perspective and preferences of PWH-OOC and other key clinical and community stakeholders on different care models in the context of the Southeastern US. In this study, we aimed to examine the relative preference and perceived advantages and disadvantages of various community-based and alternative HIV care models among PWH-OOC and other key community stakeholders.

## Methods

### Study overview

From June 2019 to May 2021, we conducted a mixed-methods study composed of structured surveys examining relative preferences of PWH-OOC for various HIV care models and qualitative in-depth interviews with PWH-OOC and other key stakeholders, to examine their perceptions of the advantages and disadvantages of the different models. We used a convergent parallel design [31], which collects the qualitative and quantitative data simultaneously and weights both methods equally.

### Study population

For the surveys with PWH-OOC, eligible participants were: 1) ≥ 18 years of age, 2) fluent in English, 3) carried an established diagnosis of HIV, 4) had not received HIV primary care within the past 6 months, and 5) had an HIV viral load over 200 copies/ml. This criteria was selected to explore the perspectives of those were truly “out-of-care,” recognizing the importance of understanding *their* preferences and needs to inform the EHE strategy of “finding effective ways to re-engage the estimated 250,000 individuals who are aware of their infection but not receiving HIV care and treatment.” Potential survey participants were identified through a Ryan White-funded HIV clinic “retention list” of OOC patients, and by direct referrals from HIV social workers and medical providers.

In-depth interviews utilized purposive sampling of an array of key clinic and community stakeholders representing the following groups: PWH-OOC, administrators, staff, providers, medico-legal compliance and billing officers, and community advisory board (CAB) members of a Ryan White-funded urban HIV clinic, city regulatory officials, staff of local AIDS service organizations (ASOs), and staff of existing mobile health clinics (MHCs) providing HIV-related services. Participants from these categories were selected out of recognition that each brought a unique lens to the perceived effectiveness, feasibility, and acceptability of the different care models based on their role. For example, PLWH-OOC could speak from a beneficiary perspective, RWC providers, staff, administrators, and CAB members brought unique dual understanding of local patient population and provider/staff needs and knowledge of clinic and program operations, clinic billing and compliance officers brought knowledge of legal and financial/billing considerations for each model, city officials brought knowledge of city regulations, permits and resources necessary to host community-based models, local ASOs brought the community perspective and knowledge of community resources that could be leveraged for the various models, and staff of existing MHCs from other contexts brought understanding of the implementation challenges and facilitators from implementing mobile services in other contexts.

For the in-depth interviews, PWH-OOC were identified using the same methods as described for the surveys above. ASOs were Atlanta-based and selected to represent a wide range of services (i.e., HIV advocacy and awareness, substance misuse and mental health counseling, nutritional services) and populations served (i.e. gay/bisexual men, cisgender women, transgender women, and under- and uninsured patients). MHC staff included those providing HIV-related services in established MHCs in the US (i.e., HIV/STI testing, linkage, and preventive services) and internationally (i.e., HIV treatment in sub-Saharan Africa). Participants were recruited through the clinic newsletter, phone, email, and in-person contact.

### Data collection

Patient participants were invited to participate in the survey, with a subset being selected for follow-up in-depth interviews. All other categories of participants only completed the in-depth interview. Surveys took place in private clinic or hospital room settings. In-depth interviews were conducted in a private site of preference to the participant (i.e., clinic space, hospital room, office). Initially, surveys and in-depth interviews were conducted primarily in person, but the COVID-19 pandemic began during enrollment. Thus, protocols were modified to allow for surveys and in-depth interviews to be conducted in a virtual environment via Zoom, phone, or other video-conference platforms. Prior to study participation, participants were informed of the potential risks and benefits associated with their involvement in the study and either written informed consent or verbal consent was obtained and documented by study staff.

Surveys were administered one-on-one by trained staff using Survey Gizmo (now Alchemer). The average survey duration was 30 minutes. Seven HIV care models were presented to the participant: 1) a mobile HIV treatment clinic, 2) telemedicine, 3) primary care clinic (whereby a patient would be provided HIV care by a primary care provider in consultancy with an offsite infectious diseases (ID) physician), 4) HIV treatment at a housing shelter or transitional housing facility, 5) HIV treatment at a drug treatment center, 6) a community-based peer navigator (who would accompany the patient from home to the existing fixed HIV/ID clinic), or 7) a fixed HIV/ID clinic (the present standard of care). For each model, participants were read a description of the model, presented with a representative image of the model (see appendix), and asked to summarize the model in their own words to confirm comprehension of the model. Example survey items that followed included *“How likely do you think patients who have fallen out of care would be to use a mobile HIV treatment clinic for their care*? After hearing about all community-based options, participants were asked to *“rank the [care models] in the order in which [they] think [they] would be likely to use them*.*”* Participants who completed the survey received $25 compensation.

In-depth interviews were audio-recorded and conducted one-on-one by trained study personnel using a semi-structured interview guide. The interview guide was tailored to participant role (i.e., care provider, patient, compliance officer) and included open-ended questions such as *“Please describe the ideal way for you to get HIV care*,*” “Why are these things [considerations] the most important for you*?*”* and *“What barriers would you face to delivering care [via xx method]*?*”* The average interview duration was 60 minutes. Those who completed an in-depth interview received $50 compensation.

### Data analysis

Survey data was collected and stored using SurveyGizmo. All quantitative analysis of survey responses was conducted using SAS statistical software version 9.3 (SAS Institute, Cary NC). Descriptive statistics of the sample’s demographic characteristics were calculated. A 3-point Likert-type scale was applied to the quantitative assessment responses (“Very likely”, “Somewhat likely,” and “Not likely”) and the distribution of responses for each item was examined. For each of the seven care models, the number of participants who ranked the model first through seventh, or not at all, was calculated. The average ranking (one through seven) was calculated for each model, excluding participants who did not rank that model.

In-depth interviews were transcribed verbatim. Transcripts were checked for quality and identifying information was removed prior to coding. The research team developed the interview codebook using deductive codes extracted from interview guides and inductive codes emerging from the interviews. Two study team members independently coded each interview using MAXQDA Plus 20.0.4 software and discussed discrepancies in coding until reaching consensus. Coded interviews were then thematically analyzed to examine stakeholder definitions of “ideal” HIV care, advantages and disadvantages for the various HIV care models, and suggestions for integrating different HIV care models.

### Ethics statement

All study procedures were reviewed and approved by the Emory University Institutional Review Board and Grady Research Oversight Committee (Approval number: IRB00109937). Study team members received training in the ethical conduct of research.

## Results

### Participant characteristics

Among the 50 survey participants, the average age was 45.5 years (σ = 12.6 years), 76% (38) identified as cisgender men, 86% (43) were Black, and 56% (28) identified as sexual minorities (Table 1). Eighty percent (40) were employed and one-third (15) were homeless or unstably housed at the time the survey was conducted. On average, participants had spent 8 nights (σ = 27.5 nights) in an overnight shelter and 38 nights (σ = 69.5 nights) on the street without shelter in the preceding six months. The average period of time living with a diagnosis of HIV was 15.8 years (σ = 10.9 years). The most common forms of payment for HIV care were Medicaid (34% or 17) and the Ryan White Program/AIDS Drug Assistance Program (34% or 17).

**Table 1:** Population and demographic characteristics of survey participants (out-of-care people living with HIV; n = 50).

**Age**	**Mean (std.)**
	45.5 (12.6)
**Sex Assigned at Birth**	**n (%)**
Male	41 (82)
Female	9 (18)
**Gender Identity**	
Cisgender Female	8 (16)
Cisgender Male	38 (76)
Transgender	2 (4)
Gender Fluid/Gender-Nonconforming	1 (2)
Other	1 (2)
**Latino/Hispanic**	
Yes	5 (10)
No	45 (90)
**Race**	
Black/African American	43 (86)
White	2 (4)
Asian/Pacific Islander	1 (2)
Other	3 (6)
None	1 (2)
**Sexual Orientation**	
Heterosexual/Straight	22 (44)
Gay/Lesbian/Homosexual/Same Gender Loving	20 (40)
Bisexual	6 (12)
Prefer to self-describe	2 (4)
**Highest Level of Education**	
Less than High School	15 (30)
High School Diploma/GED	16 (32)
Education Beyond High School	19 (38)
**Annual Household Income**	
<$10,000	32 (64)
>$10,000–19,999	13 (26)
Decline to answer	5 (10)
**Employment Status**	
Employed, Full-time	5 (10)
Employed, Part-time	5 (10)
Unemployed	40 (80)
**Payment Method for HIV Care**	
Medicare	7 (14)
Medicaid	17 (34)
Private Insurance	7 (14)
Ryan White Program/AIDS Drug Assistance Program	17 (34)
Other	5 (10)
None	3 (6)
**Housing Status**	
Homeless or unstably housed	15 (30)
	**Mean (std.)**
Nights in shelter in past 6 months	8 (27.5)
Nights on street (unsheltered) in past 6 months	38 (69.5)
**Years Living with HIV Diagnosis**	
	15.8 (10.9)

The 41 qualitative in-depth interview participants included 5 PWH; 14 Ryan White clinic providers (i.e., physicians and advanced practice providers), staff (i.e., nursing staff, mental health staff, social workers, peer navigators), and clinic administrators; 7 staff members from ASOs (that provide primary HIV care, advocacy, and preventive services); 6 CAB members; 5 staff members of domestic and international mobile health clinics (that collectively provide health services, harm reduction, and HIV testing, counseling and care); and 4 city legal and regulatory officials (i.e., city attorney, medico-legal compliance officers, and Ryan White program manager). Additional demographic information was not collected to preserve confidentiality and prevent identification given the small organization staff sizes.

### HIV care model preferences

**Fig 1:**
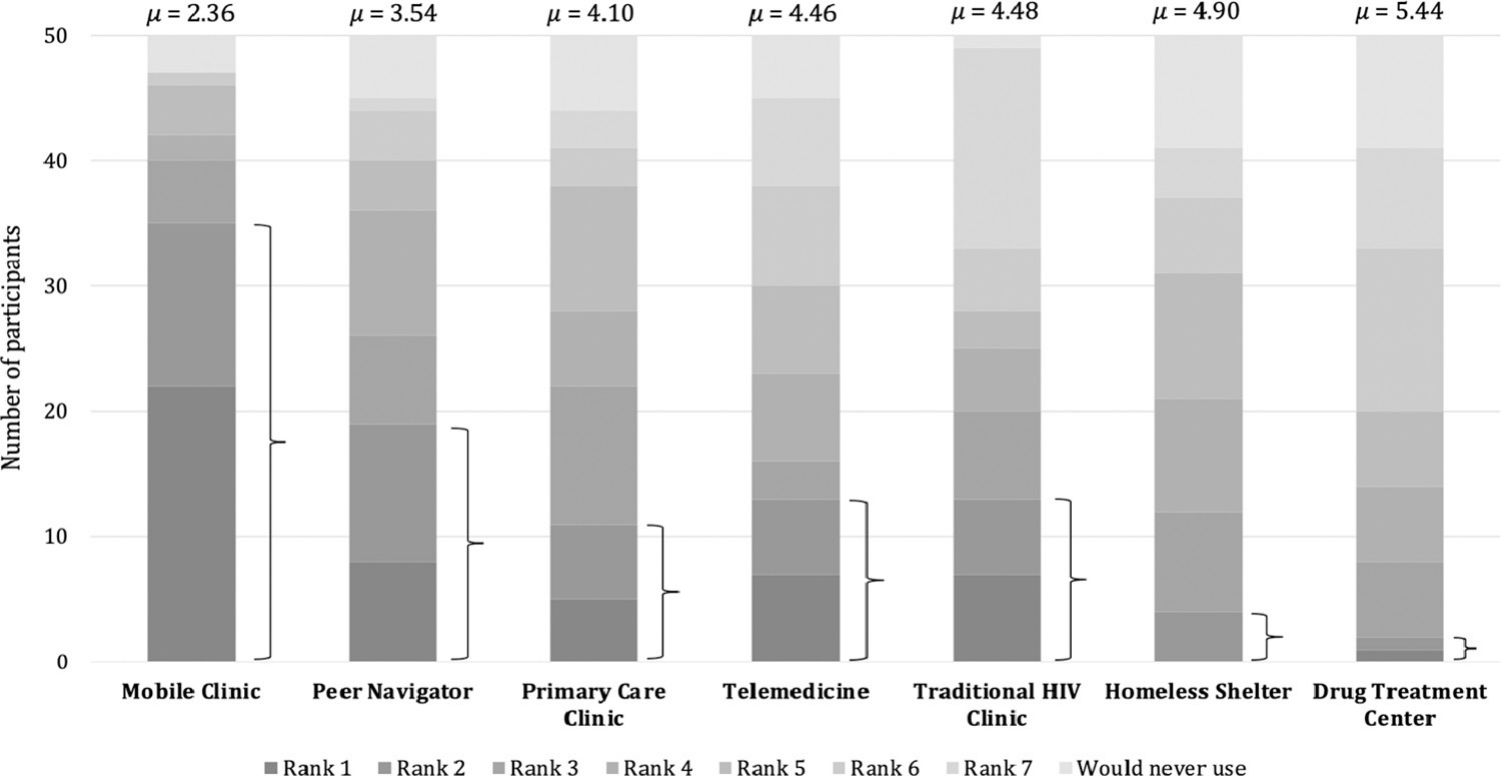
Relative preferential ranking of HIV care delivery models by out-of-care people living with HIV (PWH-OOC; n = 50). Shaded stacks represent number of PWH assigning respective rank to each of the seven care delivery models in the order they would be most likely to use them for their own HIV care. Lightest shaded stack represents number of participants who would never consider the respective model for their personal HIV care. Mean relative ranking across all participant responses was calculated for each model. Brackets indicate the number of participants who ranked each care model as their first or second choice.

Among the 50 PWH-OOC, the MHC model was ranked highest on average (μ = 2.36, σ = 1.78), followed by community peer navigators (μ = 3.54, σ = 1.95), primary care clinics (μ = 4.1, σ = 1.93), telemedicine (μ = 4.46, σ = 2.15), traditional HIV subspecialty clinics (μ = 4.48, σ = 2.30), homeless shelters and transitional housing facilities (μ = 4.9, σ = 1.66), and drug treatment centers (μ = 5.44, σ = 1.61), respectively **(Fig 1)**. Nearly 75% of participants ranked the mobile clinic model as their first or second preference. Homeless shelter and drug treatment center care models were the least preferred care sites.

Of the 50 survey participants, 76% (38/50) said they would be “very likely” or “somewhat likely” to use the mobile HIV clinic, 92% (46/50) to use peer navigation, 82% (41/50) to use telemedicine, and 82% (41/50) to use HIV care provided in primary care clinics. Of the 50 participants who were asked about receiving HIV care in shelters and drug treatment facilities, 38% (19/50) and 46% (23/50) respectively stated this did not apply to them, as they had not used these services. Of 31 participants who indicated they have stayed in shelters, 90% (28/31) said they would be very or somewhat likely to utilize HIV care provided in conjunction with temporary sheltering. Of 27 participants who have utilized drug treatment centers, 89% (24/27) said they would be very or somewhat likely to access HIV care services in this setting.

### Qualitative findings

Table 2 summarizes the perceived advantages and disadvantages of the various HIV care delivery models as reported across stakeholders.

**Table 2:** Perceived advantages and disadvantages of community-based HIV care models across HIV care stakeholders (n = 41 key informants).

Model	Advantages	Disadvantages
Mobile HIV Care	• Eliminates transportation barriers • Enables patient-centered care • Potential increase in access to other health and social services • Potential to reach more OOC individuals • Enables private, efficient medical care	• Cost • Stigma may deter utilization • Concerns for safety and security of the van/staff • Potential difficulty with troubleshooting technological or equipment malfunction in the community • Unacceptance of mobile care in some communities • Potentially restrictive rules/regulations around parking in certain communities
Community-based Peer Navigation to HIV Care	• Capacity to provide support in navigating barriers to care • Builds rapport/interpersonal connection • Reaches people at various stages of care and care readiness • Increases self-efficacy and empowers individuals to navigate HIV care • Eliminates transportation barriers • Allows for more intensive support outside of traditional clinic hours • Assists with poor health literacy • Facilitates warm transfers to other support/healthcare services • Existing evidence supporting effectiveness • Potential to integrate with other care models	• Cost/Cost Effectiveness • Building rapport/trust in community can be challenging with PWH-OOC • Potential for crossing professional boundaries • Concerns regarding high case loads • Safety concerns • Patient may exert less responsibility with their care by relying heavily on peer navigator
HIV Telemedicine	• Convenience • Eliminates transportation barriers • Potential to reduce stigma by maintaining privacy/confidentiality in one’s personal space • Ease of implementation given high number of mobile phone users • Safe • Capacity to engage younger populations in care • Capacity to expand care to rural and other underserved areas • Ability to incorporate multi-disciplinary services	• Logistically challenging • Lack of adequacy of required resources (i.e., phone, internet access) among patient population • Potential costs • Possible decrease in quality of care as the provider’s assessment cannot incorporate a full physical examination • Potential usability challenges for individuals with poor technological skills
Homeless Shelter or Drug Treatment Center-based HIV Care	• Convenience • Discreetness • High likelihood of reaching priority population as many PWH also experience housing instability and substance abuse • Eliminates communication barriers through ease of access to knowledgeable staff members • High feasibility and low cost due to ease of integration into existing services	• Challenges with finding physical space • Potential difficulty with maintaining privacy and confidentiality • Could enhance HIV stigma • Adherence Barriers (Homelessness, mental health, substance use, etc.) • Patients (shelter residents) likely to have other competing needs/stressors affecting prioritization of HIV care
Community-based Primary Care with ID Physician Consultancy	• Low cost, high feasibility • Convenience • Discreetness • Capacity to provide comprehensive HIV-related and non-HIV-related care	• Requires training in HIV care practices for primary care providers • Requires the establishment of a relationship between the primary care provider and ID specialist • Limited time of primary care providers • Many PLWH-OOC lack established primary care providers or lack trust with primary care providers

### Mobile HIV clinic

#### Advantages

Several participants discussed convenience as a central benefit of the mobile clinic.

*“Knock on that door and say ‘we’re right down the street*, *we’re right here*, *you don’t even have to put on outside clothes*, *come in your pajamas*, *you know*, *we’re right here’ … at least we’re making it a little bit easier to get the care that he needs*.*”–*Nursing Staff

*“I’m thinking it will work out really good for people who don’t really feel like really being away from the home that long*, *because sometimes going to the doctor…it’s an all-day event and it’s really tiresome*.*”*–Patient

Many discussed that the mobile clinic could have greater reach by addressing and reducing barriers to care like physical disability, housing insecurity, inadequate access to transportation, and patient unwillingness to travel to a clinic.

*“Our patients that have problems with*, *you know*, *homelessness*, *home insecurity*,*…physical difficulties*, *they have a MARTA [public transportation] card but they have horrible neuropathic pain in their feet*, *so being able to like walk a block*, *rather than walking all the way to MARTA*, *getting off of MARTA*, *trying to make your way into the hospital*.*”*–Provider

*“I think [the mobile clinic] will help a lot of people who just don’t want…to come to the doctor’s office*.*’”*–CAB Member

An ASO staff member also discussed that the mobile van could serve as a visual reminder to individuals to seek care:

*“I think a lot of people just driving past be like*, *‘Oh*, *maybe I should stop in*.*’ Even if it wasn’t on their mind*, *even though they hadn’t seen all of the internal communication*, *but just it being present out in the community instead of*, *kind of*, *I guess*, *at the hospital off at a distance*. *It’s like right there in the middle of everything*.*”–*ASO Staff

Lastly, some talked about the potential of the MHC to reduce HIV stigma in the community as an advantage of the model.

*“Make it more comfortable*, *and I feel like*, *you know*, *more frequent presence in the community*, *you know*, *become more familiarized with us*. *So*, *that kind of stigma*, *you know*, *the stigma is reduced*.*”*–Mobile Unit Staff Member

#### Disadvantages

Many raised concerns over safety of staff and others on the mobile clinic as a potential disadvantage.

*“What would you do about security*? *Um*, *there’s you know*, *there’s*, *there’s a lot of um hateful people and you know*, *so you know those are some concerns I have*.*…I’d hate for any clinic to be a target of*, *uh of*, *you know*, *any kind of violence or hatred”–*CAB Member

Other potential disadvantages raised included the ability to maintain confidentiality and not contribute to HIV stigma, as well as the possibility that care on the bus could feel impersonal.

*“They would have to camouflage it…I know it would make me feel uncomfortable if the whole town knew that’s what it was*.*”*–Patient

*“No matter how you design it*, *after a while it’s*, *‘That’s the HIV bus*.*’”*–Social Worker

*“Cons would be*, *who are these people*? *Uh does it feel like I’m receiving care from a stranger*?*”–*ASO Staff

### Community-based peer navigator

#### Advantages

A key advantage of the community-based peer navigation model is its compassionate approach in providing patients a “friend” to help navigate the complex health system.

*“Just leaving a sticky note*, *you know*, *‘Mike stopped by*,*’ you know ‘just checking on you*,*’ and that maybe makes people say*, *‘Hey*, *somebody cares about me here*,*’ and that may make them reach out and say*, *‘I need to take care of this*,*’ you know*. *Gives them accountability*, *I think…I think just having that extra voice out there*, *someone concerned…When I was so sick*, *just trying to maneuver the system was very difficult*, *and I think just how much better would it have been if someone had been able to reach me through my phone*, *uh*, *through that type of telemedical services or could come to my house*.”–Patient

*“Having a human being that they can*, *you know*, *become friends with*, *I think with our population it’s all about establishing a relationship*. *And if they know this person*, *know how to reach them*, *this person knows where*, *you know*, *you can typically find them and actually get them to the clinic*, *phenomenal*.*”*–Provider

“*I think it just connects people to the system*. *Like*, *it’s so easy to get lost in the system*. *Like*, *just from*, *like*, *just like*, *the first step*, *of like*, *finding parking–or not even–that’s not even the first step*. *Like*, *literally getting in–getting on MARTA*, *to like*, *come here as a first step and then*, *like*, *finding parking or whatever*. *Like there’s so many*, *like*, *unique challenges along the way and then finding this clinic*, *like*, *filling out your paperwork; ‘Where is the lab*?*’; going to the lab knowing that*, *‘Oh*, *like they want to do a chest x-ray*, *also*.*’”*–Provider

Participants also discussed that a peer could better understand and advocate for patient needs.

*“And I think that a lot of people just really need support*. *I don’t think it’s so much of money and food*. *I think they just need support*, *you know*, *just to let them know that if there’s anything you could do or if it’s anything I need to speak on or be their advocate–because people need that*, *because sometime*, *like a lot of times*, *I can’t really say what I want to say*, *so if I have an advocate there they understand where I’m trying to come from*.”–Patient

*“Yeah*, *you’re meeting people where they are*, *you’re meeting them in their home setting*. *You may*, *you know*, *walk up and see*, *you know*, *a lot of the things that may be preventing them from*, *you know*, *from getting there*. *You know if the house is awry and you know there’s screamin’ and yelling* …*the peer navigator can pick up on a lot of things like that*.*”*–Provider

A provider further elaborated that a peer’s informed understanding of a patient’s needs and effective communication of those needs could additionally enhance the efficiency and effectiveness of clinic visits.

*“It would make our visits more productive…There would be sort of less need for explanation or rehashing some basics…It would just like free up time*, *and it would probably allow patients to sort of ask higher questions…having a navigator speak for them in a way that maybe if the patient was sort of like not wanting to ask*.*”–*Provider

Others discussed that peers are trusted and facilitate patient comfort.

*“I think people trust their peers more than a provider in a coat*, *or for us*, *too*. *I think people are a little leery that we look different from them*. *We’re young*, *pretty ladies*, *and we just don’t look like them*. *And I think they’re hesitant to work with us sometimes or open up*. *So*, *I think peers are people that others trust*, *and they’re knowledgeable about the systems that are going on*, *too*.”–ASO Staff

*“A navigator is*, *I think is real good for someone in the beginning stage who just found out*. *Especially like the young kids here…they need someone to talk to–besides a friend—that may be positive as well*.*”*–CAB Member

#### Disadvantages

Participants raised privacy and confidentiality concerns with the peer model.

*“The one thing that I worry about is*, *um*, *of course getting their consent*, *making sure that*, *you know*, *this is*, *like*, *how do you handle privacy issues*, *a lot of my patients live with other*, *you know*, *family members and friends*, *some know their HIV status…some don’t*.*”*–Provider

*“I could probably go into a Black neighborhood and maybe not be noticed*. *If I’m Caucasian or Asian or Hispanic*, *‘Who’s that man that be in the neighborhood*?*’ You know neighborhoods talk*, *because people*, *homeowners*, *want to know who you are…If I just drove into your house that may be different*. *But if I’m seeing multiple patients in that area*, *people want to know who you [are]*.*”–*Peer Navigator

Some questioned the overall effectiveness of the peer model in addressing patient barriers and the overextension of the peer role.

*“The barriers would be just*, *um*, *patients not sticking to what they’ve agreed that they said they would do*. *So*, *you have this expectation*, *for an example*, *the peer navigator goes out and he has an expectation to meet*, *um*, *this patient at this time*, *another patient at this time*, *and another patient at this time*, *and it’s all planned out*, *because they have been communicating with these patients and the patients have agreed to be there*. *And they don’t show up*, *they’re not answering their phones*. *So*, *barriers would still be*, *I think*, *the patients themselves*. *Um*, *what we have to offer is*, *is there*. *But one thing we can never control is the patient’s willingness*.*”*–Social Worker

An additional disadvantage discussed by a provider was that the success of the peer model would be heavily reliant on the strength of provider-peer communication, which can be difficult given time constraints within the health system.

*“I think that it would take a lot of communications between like a navigator and the provider though*, *and it is hard getting a provider with time constraints…Like saying*, *‘Hey like*, *you’re going to see a patient who I’m helping tomorrow*. *Just so you know*, *like*, *here are some issues I’ve identified’…*.*We get flooded with communication a lot*, *but it probably would be*, *like*, *a high-yield communication–prior to a clinic visit*.*”*–Provider

Lastly, concern was raised about the use of the peer model leading to patient perceptions that others do not see them as capable of adhering to care.

*“You don’t wanna make people feel like*, *‘We don’t think you’re going to be responsible so*, *you know*, *we are going to assign somebody to help you out*, *get you through this*.*’ I don’t want to be made to feel like I’m not gonna be responsible*.*”–*CAB Member

### Primary care clinics

#### Advantages

Convenience was raised as an advantage for using primary care clinics to deliver HIV care along with remote HIV consultation.

*“If it cuts down on my*, *my travel time and*, *you know*, *if it just makes everything more convenient*, *that’s great*.*”–*CAB Member

Primary care clinics could be particularly useful for patients without acute care needs who simply require routine monitoring while on antiretroviral therapy.

*“I think those who are stable and*, *like*, *don’t need to come to […] their [HIV] provider*, *um*, *every 3 months*, *would really be good candidates for primary care*. *Those*, *I mean*, *they’re not sick*, *they’re pretty much just need to be maintained*, *have that prescription refilled every so often*, *make sure their viral load is suppressed*.*”*–Nursing Staff

Primary care clinics were also seen as having the potential to provide more comprehensive, less-stigmatized care. An ASO staff member explained this advantage through a case study of a patient who would not attend a traditional HIV clinic.

*“He would never have gone to the*, *to an AIDS clinic in the beginning*, *but that also allowed them to address some of his other social issues that were barriers for him*. *So*, *he needed that holistic care*. *He had other medical problems as well*. *So*, *I think that’s a great idea to wrap that into what people see as more regular healthcare*.*”*–ASO Staff

Participants discussed how primary care clinics may be more familiar, comfortable settings for patients.

*“You’re already there*, *they’re already comfortable*. *They’re in the setting that they know*, *and they trust what’s going on there*, *so they’re gonna trust that what we’re doing*.*”*–Nursing Staff

#### Disadvantages

The time limitations of routine primary care visits were raised as a potential disadvantage of this model.

*“I think a lot of primary care providers*, *you’re talking about short period of times seeing patients*, *they have 15 to 20 minutes time*, *so if they can go to a primary care clinic*, *I would say come to our clinic where you’ll actually have*, *um*, *more support*.*”*–Provider

Another barrier raised was that there would be limited HIV expertise at the primary care clinics and that quality of care for patients may suffer as a result.

*“I know that primaries can do a lot of things*, *but I think HIV—if you do it*, *live it*, *breath it—you’re better at it*. *That’s just how I feel*.”–Peer Navigator

*“I might fear that the*, *uh*, *that the service will not be on par to what [the HIV/ID clinic] is*.*”*–CAB Member

Others raised the concern that patients may not have established primary care providers due to disparities in primary care access or because patients perceive that primary care provision lies within the scope of HIV care clinics.

*“I see gaps and disparities in access to primary care*, *and I*, *you know*, *I know that that’s an issue*. *So*, *I*, *you know*, *I worry that some people who don’t get–who traditionally don’t get HIV care also don’t get primary care*, *so I worry about them*.*…the people who are most at risk for HIV are also some of the most marginalized from mainstream healthcare*. *So*, *I don’t think we’re going to get to a really good place just with that*.*”*–ASO Staff

*“I don’t know about primary care clinics*, *only because a lot of our patients think that we’re primary care*.*”*–Social Worker

Participants also raised concerns of patient-provider trust being a disadvantage with seeking HIV care at primary care clinics.

*“Some people may not always be insured*, *or they are maybe uncomfortable talking to their primary care doctor about HIV*. *And you know*, *often times*, *… I don’t know the data behind this–but there is this barrier of people who are of the LGBT community with speaking to primary care doctors about things that’s related to sexual health*.*”*–Mobile Unit Staff Member

### Telemedicine

#### Advantages

Many participants discussed convenience, privacy, and comfort as key advantages of telemedicine.

*“It’s definitely a little bit more private*. *It’s quick access*, *so–and you’re more comfortable*, *you know–you don’t have to be seen by other people; you don’t have to you–you know*, *you just have*, *like*, *the privacy of your own home*, *so I definitely think that’s–it’ll be great and it eliminates those barriers and eliminates*, *you know*, *you being influenced by stigma*, *because you don’t have to worry about going to a clinic*, *being seen*, *or what others might think that*, *you know*, *you are getting your HIV treatment*.*”*–Mobile Unit Staff Member

*“It’s makes it especially appealing for people with mental health*. *Some people don’t want to go outside*, *don’t want to be bothered*, *see what other people*. *So*, *this will make them more comfortable*.*”–*CAB Member

Some discussed that telemedicine could cater to younger populations and that it aligns with current tech culture.

*“I think that’s an awesome idea for people who do*, *who are computer savvy and have*, *um*, *internet access*, *probably the younger generation*, *generation X and the millennials*, *would love that*. *They don’t have to*, *‘I don’t have to come in*, *I could still get treatment*.*’ Oh*, *my god*, *they would still love this*, *because it’s not really an interaction*.*”*–Nursing Staff

*“I just think that people live by their phones and people live by Instagram*, *and I mean*, *I think if they agree to it we could send them reminders that way*. *Just communicating with them*, *they live by their phones*.”–Peer Navigator

One patient discussed that it could be used as a tool to convince patients of the need to physically come into the clinic.

*“It would probably make it a hell of a lot easier to convince your patient*, *as they come into the clinic*, *when it’s really necessary to come in and get checked out*.”–Patient

#### Disadvantages

While some participants argued that everyone has a phone, others spoke of potential barriers, including lack of access to phones, telemedicine technology, and data, cost, and technology literacy, as major disadvantages of this model.

*“A lot of times*, *our patients that are falling out of care are*, *um*, *housing insecure*, *and when you’re housing insecure*, *a lot of times you’re cell phone insecure*. *And a lot of times*, *my patients who have cell phones*, *they don’t know how to do this fancy stuff*. *Some of mine have a*, *a little flip phone*, *so I kind of wonder how many patients would actually benefit from this*.*”*–Provider

CAB members further raised potential challenges with technology and coordination logistics as disadvantages of telemedicine.

*“We do know with computer technology shit*, *stuff does go down*. *We know that*, *you know what I’m saying*, *but why is it every time that I’m getting on this app*, *I’m not able to see*, *I’m not able to read anything*, *it just–it just says ‘App is down*.*’”*–CAB Member

*“But when you’re talking about doctors*, *you have to have willing doctors that’s willing to be on there for a certain time to say*, *‘I’ma have patients*.*” But then*, *if*, *let’s just say*, *they’re on there [the telemedicine platform] for eight hours and nobody comes on there*, *you know*, *that’s gonna kill that*.*”*–CAB Member

Some raised concerns of control over privacy and confidentiality of a telemedicine visit.

*“They might not even be in a private place to be able to do it*.*”*–CAB Member

*“You can see that that is just one person on*, *on the line with you*. *And now if you do have other people around you of course the person*, *uh*, *the professional has no control over that*.*”*–Social Worker

A social worker discussed the reduced ability to handle mental health emergencies via a telemedicine visit.

*“Mental health is always tricky–sorry–when it comes to telemedicine*, *because*, *um*, *you know*, *what if the patient’s in crisis or on the brink of suicide*? *I think that puts us in a very interesting position*.*”*–Social Worker

One participant discussed that telemedicine may inadvertently provide the message that the clinic perceives the patient as being helpless and having low self-efficacy.

*“The unwitting message that might be sent to the patient is ‘You are incapable of coming in*, *right*? *So*, *so you’re fragile*, *so we need to*, *um*, *we need to make sure you don’t leave and that we sort of attend to you in this way*.*’”*–Mental Health Clinician

### Housing shelters and drug treatment centers

#### Advantages

Many participants discussed that provision of HIV care at temporary housing shelters and drug treatment facilities had the advantage of providing convenient, comprehensive, and one-stop integrated care.

*“A lot of our patients do struggle with homelessness*, *and so*, *if you had*, *at*, *at these facilities*, *not just the medical care*, *but somebody to help with–um*, *you know*, *get them on the*, *linked with the resources they would need for*, *for housing…if you want to provide holistic care*, *it’s not just the medical piece that has to be addressed*.*”*–Social Worker

*“When I worked in HIV care*, *the other thing that really helped people with access to substance abuse treatment [was not having] to choose between that and their HIV care*, *but the integration of drug treatment programs with their HIV care*.*”–*ASO Staff

An additional benefit of these models is their capacity to reach people by ‘meeting them where they are,’ in spaces they trust and are comfortable with.

*“For the shelters*, *I like the idea of meeting them where the need’s at already*. *They’re already there*. *They’re already comfortable in those situations*, *and then having that relationship with another facility…they trust what’s going on there*, *so they’re gonna’ trust that what we’re doing*, *and we could provide that need [HIV care] for them there*.*”*–Nursing Staff

Concomitant treatment services within this model could enable patients to reflect over the intersection of HIV, stigma, and drug use and foster their efforts to overcome each.

*“And so*, *when they’re getting clean*, *their thought processes are changing*. *And they’re in a place where they have counselors*, *and … that may be one of the underlying issues to why their drug behavior is the way it is*, *is because of living with HIV and the stigma and the shame associated with it*. *So*, *they*, *they may have used or use drugs more to try to deal with it*. *So*, *I think to have someone there*, *maybe that could assist them with knowledge about HIV and the importance of staying in care*, *and you know*, *uh*, *while they’re in a counseling setting would be really well*.*”–*Patient

The potential for reaching vulnerable populations was also raised as an advantage of shelter-based care.

*“There are lots of intravenous drug users that are living in transitional housing right now*. *Lots of transgender population are living in transitional housing*, *and in transitional housing comes lots of survival sex*. *And survival sex–‘I need money; I need food; so I’ll have sex with you*.*’*–Peer Navigator

Lastly, a participant highlighted that these models may reduce HIV stigma by integrating HIV care into existing primary care at these facilities. They discussed, in the context of people experiencing homelessness, the common occurrence of foot wounds:

“*It can’t get much more normalizing than where you can take care of your feet and your HIV at the same time*.*”–*ASO Staff

#### Disadvantages

Inability to maintain confidentiality was seen as a critical disadvantage of providing HIV care at shelters and drug treatment facilities, although some considered this challenge to be surmountable.

*“No matter … how well placed in the shelter a clinic might be …*, *some people in the shelters don’t come out*, *because they don’t want nobody in the shelter to know*.*”–*ASO Staff

*“People still don’t want people to know that ’If I go down that left hallway*, *that’s where the HIV people are*.*’ But if you could kind of mix it in with all the other services that are offered*, *then I can see that*.*”*–Peer Navigator

Some raised concern about the reach of HIV services in drug treatment shelters given low attendance of substance abuse treatment and that providing HIV care at a drug treatment shelter may inadvertently detract a patient’s focus from substance abuse treatment to HIV care.

*“I wasn’t crazy about [HIV care at substance abuse treatment centers]*, *because*, *to be honest with you*, *I feel like there’s a limitation to getting patients into a drug treatment facility in the first place*.*”–*Provider

*“I think that if a person’s in a drug treatment center*, *that should be their main focus and not their HIV drugs*.*”*–Peer Navigator

#### Additional innovative and combined models of community-based HIV care

Several participants discussed the care models as complementary, each serving unique patient needs and preferences, and discussed possibilities of merging them.

*“I mean*, *ideally*, *it’s probably a combination of all of these*.*”–*Program Manager

*“I think sort of…the strategies that you’re describing fit different patients in different ways*, *and there are certain patients who may benefit from certain models more than other ones*. *And so*, *I’m not sure if there’s like a–you know*, *one size fits all*.*”–*Provider

A patient gave an example of using telemedicine to conduct the clinical visit and having the mobile clinic available thereafter for blood collection for labwork.

*“Well*, *I think that they could all be kinda merged together…Telemedicine and then having [the mobile unit] where it’s set up where*, *you know*, *you can go get your labs locally*. *Um*, *I think that’s a huge benefit*. *Especially if I didn’t have the money to pay for MARTA or transportation*, *um*, *you know*, *it’s 5 bucks roundtrip*. *I mean*, *for some people 5 bucks is a meal*, *and that’s just where we’re at*.*”–*Patient

Another spoke of the opportunity for the mobile clinic to be used to provide care at shelters and transitional housing facilities.

*“I think it’s a good option*, *because*, *of course*, *we can roll the mobile unit to the drug treatment center*, *and we can roll the mobile unit at the shelter and transitional housing*.*”–*Mental Health Case Manager

Others spoke of the community peer navigation model being used to aid the patient in utilizing other beneficial care models.

*“The peer navigator would be able to…offer them other ways*, *which would be … the treatment clinic*, *the mobile treatment clinic*, *or the telemedicine*, *or the primary care*, *or you know any of the other things*.*”–*Social Worker

An ASO staff member corroborated the utility of the merged community navigator approach through her prior experience.

*“It seems like the peer navigators need to be in the community doing outreach*, *and this was a model that was happening in Chicago where I was*. *So*, *I saw it firsthand*. *They were out in the communities*. *They’d go to some of the encampments or just the neighborhoods that had a higher risk of transmission*, *and they built those relationships*, *and then brought them along*. *Then*, *people started coming into the clinics*, *and it was really wonderful to see*. *It didn’t work every time*, *but it was—I think it was more helpful than that people have this familiar face to work with*. *So*, *peer navigator’s in the community doing outreach*, *and then during those sessions*, *then they’re like*, *‘Oh*, *did you know this mobile van is going to be in this place at this time*? *I’ll be there on Tuesday*. *Come meet me*.*’ Or ‘Did you know we had telemedicine where you could talk to your doctor over [the phone]*?*’”–*ASO Staff

Lastly, a few patients raised novel models of care that were not presented in the interview guides. One idea was establishment of a community center that provided non-HIV care alongside HIV services and support services. A second was using a phone app to triage patients based on their needs.

*“Cuz’ you got people that may be in a situation where they need housing*, *so if you could connect someone with*, *you know*, *there’s so much that you could do with an app…you know*, *for housing–‘Click here*,*’ for drug counseling–‘Click here*,*’ for case management–‘Click here*,*’ for peer coordinator–‘Click here*,*’ for doctor–‘Click here*.*’ You know*, *to have access to all of those things and got a response that said within 24 hours*, *‘We’re working on this and we’re going to get back to you*.*’”–*Patient

## Discussion

Innovative approaches to HIV care delivery have the potential to revolutionize HIV treatment services for PWH-OOC by providing care in more accessible, convenient, and patient-centered ways. To our knowledge, this is the first mixed-methods study exploring relative preference for community-based and alternative HIV care models among PWH-OOC and advantages and disadvantages to implementation of each model among key community stakeholders. Importantly, a significant proportion of the PWH-OOC who participated in this study were racial and sexual minorities, of low socio-economic status and unstably housed, and living the US South–critical subgroups whose care preferences are rarely heard and included in the design of retention programs. Had this study been conducted in a general population of PWH, the care preferences identified may have been vastly different. Further, the preferences and perceived advantages and disadvantages differed across patients and other key stakeholders in our study, underscoring the importance of including these diverse perspectives in future studies that pilot these models and examine the factors influencing their acceptance and feasibility.

The highest preference for care delivery in this study was via the mobile clinic, followed by community-based peer navigation, primary care clinics, telemedicine, traditional HIV subspecialty clinics, homeless shelters, and drug treatment centers. These findings suggest that community models are not only acceptable, but in many cases preferable to traditional fixed clinics among PWH-OOC. Numerous prior studies support the acceptability of alternative care models for PWH [21–23,32–34], and corroborate the perceived benefits identified here of convenience, accessibility, enhanced privacy, and capacity to demonstrate compassion and foster patient-provider rapport. Interestingly, studies aiming to understand patient preferences and trade-offs for HIV care delivery using discrete choice experiments suggest that many patients are willing to sacrifice convenience and accessibility for “nice” providers and “patient-centered” care teams [35,36]. Community-based care models would not require patients to choose between these qualities, but rather have the potential to provide the best of all worlds—convenient, accessible *and* compassionate care—as HIV care delivery would be less bound by the rigidity and time constraints of fixed-clinic visits, and could demonstrate care and concern by “meeting people where they are.”

The challenges identified across the alternative care models, including loss of privacy and confidentiality, physical safety, and logistical and technical access challenges, have also been noted in part by prior studies [34], and underscore the importance of strategies addressing these concerns to be at the core of protocol design for these alternative care delivery models. Lastly, participant insistence on the need for PWH to be able to choose different care models at different times suggests that Ending the HIV Epidemic efforts should ensure high-priority jurisdictions are equipped with various community-based HIV care models in addition to traditional fixed HIV clinics for PWH to select from.

A key strength of this study was its assessment of multiple stakeholder perspectives, with dedicated efforts to ensure PWH-OOC were at the forefront. A limitation was the small sample size, which limits generalizability and potential for subgroup analyses. We also recognize that care preferences may not reflect actual behavior (use of services), thus future studies should examine relative uptake of these various models. Lastly, while non-patient stakeholders provided perspective on the advantages and disadvantages of each model, they were not asked to rank their preference for each care model. This could be explored in future research.

In conclusion, these findings highlight the need to integrate alternative and community-based care models into the national plan to End the HIV Epidemic, and to allow PWH-OOC to choose the model most fitting for their individual circumstances. Future research should focus on methods to address patient concerns surrounding the use of the different care delivery models. Additionally, future studies should explore pilot implementation of the preferred community programs (i.e., mobile clinics, community-based peer navigation) and evaluate their impact on HIV care retention and viral suppression.

## References

[1] Office of Infectious Disease and HIV/AIDS Policy, HHS. What is Ending the HIV Epidemic in the U.S.? [updated June 2, 2021. Available from: https://www.hiv.gov/federal-response/ending-the-hiv-epidemic/overview.

[2] What Is the HIV Care Continuum? [updated June 21, 2021. Available from: https://www.hiv.gov/federal-response/policies-issues/hiv-aids-care-continuum.

[3] Crawford TN, Sanderson WT, Thornton A. Impact of poor retention in HIV medical care on time to viral load suppression. J Int Assoc Provid AIDS Care. 2014;13(3):242–9. doi: 10.1177/2325957413491431 23761217

[4] Yehia BR, French B, Fleishman JA, Metlay JP, Berry SA, Korthuis PT, et al. Retention in care is more strongly associated with viral suppression in HIV-infected patients with lower versus higher CD4 counts. J Acquir Immune Defic Syndr. 2014;65(3):333–9. doi: 10.1097/QAI.0000000000000023 24129370 PMC3945404

[5] Kay ES, Batey DS, Westfall AO, Christopoulos K, Cole SR, Geng EH, et al. Compound Retention in Care and All-Cause Mortality Among Persons Living With Human Immunodeficiency Virus. Open Forum Infect Dis. 2019;6(4):ofz120. doi: 10.1093/ofid/ofz120 31041339 PMC6483128

[6] Mugavero MJ, Lin HY, Willig JH, Westfall AO, Ulett KB, Routman JS, et al. Missed visits and mortality among patients establishing initial outpatient HIV treatment. Clin Infect Dis. 2009;48(2):248–56. doi: 10.1086/595705 19072715 PMC2737584

[7] Giordano TP, Gifford AL, White AC Jr., Suarez-Almazor ME, Rabeneck L, Hartman C, et al. Retention in care: a challenge to survival with HIV infection. Clin Infect Dis. 2007;44(11):1493–9. doi: 10.1086/516778 17479948

[8] Centers for Disease and Prevention. HIV in the United States and Dependent Areas.

[9] Centers for Disease Control and Prevention. Issue Brief: HIV in the Southern United States2019. Available from: https://www.cdc.gov/hiv/pdf/policies/cdc-hiv-in-the-south-issue-brief.pdf.

[10] Centers for Disease Control and Prevention. About Ending the HIV Epidemic in the U.S. Initiative [updated September 7, 2021. Available from: https://www.cdc.gov/endhiv/about.html.

[11] Rueda S, Mitra S, Chen S, Gogolishvili D, Globerman J, Chambers L, et al. Examining the associations between HIV-related stigma and health outcomes in people living with HIV/AIDS: a series of meta-analyses. BMJ Open. 2016;6(7):e011453. doi: 10.1136/bmjopen-2016-011453 27412106 PMC4947735

[12] Katz IT, Ryu AE, Onuegbu AG, Psaros C, Weiser SD, Bangsberg DR, et al. Impact of HIV-related stigma on treatment adherence: systematic review and meta-synthesis. J Int AIDS Soc. 2013;16(3 Suppl 2):18640. doi: 10.7448/IAS.16.3.18640 24242258 PMC3833107

[13] Buckingham E, Schrage E, F. C. Why the treatment of mental disorders is an important component of HIV prevention among people who inject drugs. Advances in Preventive Medicine. 2013;690386. 23401785 10.1155/2013/690386 PMC3562640

[14] Lucas G. Substance abuse, adherence with antiretroviral therapy, and clinical outcomes among HIV-infected individuals. Life Sci. 2011;88(21–22):948–52. doi: 10.1016/j.lfs.2010.09.025 20888839 PMC3027844

[15] Goswami ND, Schmitz MM, Sanchez T, Dasgupta S, Sullivan P, Cooper H, et al. Understanding Local Spatial Variation Along the Care Continuum: The Potential Impact of Transportation Vulnerability on HIV Linkage to Care and Viral Suppression in High-Poverty Areas, Atlanta, Georgia. J Acquir Immune Defic Syndr. 2016;72(1):65–72. doi: 10.1097/QAI.0000000000000914 26630673 PMC4837075

[16] Terzian AS, Younes N, Greenberg AE, Opoku J, Hubbard J, Happ LP, et al. Identifying Spatial Variation Along the HIV Care Continuum: The Role of Distance to Care on Retention and Viral Suppression. AIDS Behav. 2018;22(9):3009–23. doi: 10.1007/s10461-018-2103-8 29603112 PMC6468992

[17] Masiano SP, Martin EG, Bono RS, Dahman B, Sabik LM, Belgrave FZ, et al. Suboptimal geographic accessibility to comprehensive HIV care in the US: regional and urban-rural differences. J Int AIDS Soc. 2019;22(5):e25286. doi: 10.1002/jia2.25286 31111684 PMC6527947

[18] Sagrestano LM, Clay J, Finerman R, Gooch J, Rapino M. Transportation vulnerability as a barrier to service utilization for HIV-positive individuals. AIDS Care. 2014;26(3):314–9. doi: 10.1080/09540121.2013.819403 23876086

[19] Andersen M, Hockman E, Smereck G, Tinsley J, Milfort D, Wilcox R, et al. Retaining women in HIV medical care. J Assoc Nurses AIDS Care. 2007;18(3):33–41. doi: 10.1016/j.jana.2007.03.007 17570298

[20] Centers for Disease Control and Prevention. HIV Testing and Treatment: Meeting People Where They Are [updated August 17, 2020. Available from: https://www.youtube.com/watch?v=7pUp0ROH5yI.

[21] Moon TD, Jequicene T, Blevins M, Jose E, Lankford JR, Wester CW, et al. Mobile clinics for antiretroviral therapy in rural Mozambique. Bull World Health Organ. 2014;92(9):680–4. doi: 10.2471/BLT.13.129478 25378759 PMC4208568

[22] Gorman SE, Martinez JM, Olson J. An assessment of HIV treatment outcomes among utilizers of semi-mobile clinics in rural Kenya. AIDS Care. 2015;27(5):665–8. doi: 10.1080/09540121.2014.986053 25495796

[23] Babigumira JB, Sethi AK, Smyth KA, Singer ME. Cost effectiveness of facility-based care, home-based care and mobile clinics for provision of antiretroviral therapy in Uganda. Pharmacoeconomics. 2009;27(11):963–73. doi: 10.2165/11318230-000000000-00000 19888795 PMC3305803

[24] Selke HM, Kimaiyo S, Sidle JE, Vedanthan R, Tierney WM, Shen C, et al. Task-shifting of antiretroviral delivery from health care workers to persons living with HIV/AIDS: clinical outcomes of a community-based program in Kenya. J Acquir Immune Defic Syndr. 2010;55(4):483–90. doi: 10.1097/QAI.0b013e3181eb5edb 20683336

[25] Bradford JB, Coleman S, Cunningham W. HIV System Navigation: an emerging model to improve HIV care access. AIDS Patient Care STDS. 2007;21 Suppl 1:S49–58. doi: 10.1089/apc.2007.9987 17563290

[26] Mizuno Y, Higa DH, Leighton CA, Roland KB, Deluca JB, Koenig LJ. Is HIV patient navigation associated with HIV care continuum outcomes? AIDS. 2018;32(17):2557–71. doi: 10.1097/QAD.0000000000001987 30102661 PMC6724743

[27] Ohl M, Dillon D, Moeckli J, Ono S, Waterbury N, Sissel J, et al. Mixed-methods evaluation of a telehealth collaborative care program for persons with HIV infection in a rural setting. J Gen Intern Med. 2013;28(9):1165–73. doi: 10.1007/s11606-013-2385-5 23475640 PMC3744312

[28] Smith E, Badowski ME. Telemedicine for HIV Care: Current Status and Future Prospects. HIV AIDS (Auckl). 2021;13:651–6. doi: 10.2147/HIV.S277893 34140812 PMC8203096

[29] Coleman SM, Blashill AJ, Gandhi RT, Safren SA, Freudenreich O. Impact of integrated and measurement-based depression care: clinical experience in an HIV clinic. Psychosomatics. 2012;53(1):51–7. doi: 10.1016/j.psym.2011.07.004 22221721

[30] Dillard D, Bincsik AK, Zebley C, Mongare K, Harrison J, Gerardi KE, et al. Integrated nested services: Delaware’s experience treating minority substance abusers at risk for HIV or HIV positive. J Evid Based Soc Work. 2010;7(1):130–43. doi: 10.1080/15433710903176021 20178030

[31] Creswell JW, Plano Clark VL. Designing and Conducting Mixed Methods Research. 3rd Ed ed. Thousand Oaks, CA: SAGE Publishing; 2017.

[32] Trujillo D, Turner C, Le V, Wilson EC, Arayasirikul S. Digital HIV Care Navigation for Young People Living With HIV in San Francisco, California: Feasibility and Acceptability Study. JMIR Mhealth Uhealth. 2020;8(1):e16838. doi: 10.2196/16838 31922489 PMC6996763

[33] Roland KB, Higa DH, Leighton CA, Mizuno Y, DeLuca JB, Koenig LJ. Client Perspectives and Experiences With HIV Patient Navigation in the United States: A Qualitative Meta-Synthesis. Health Promot Pract. 2020;21(1):25–36. doi: 10.1177/1524839919875727 31597497 PMC6917848

[34] Dandachi D, Dang BN, Lucari B, Teti M, Giordano TP. Exploring the Attitude of Patients with HIV About Using Telehealth for HIV Care. AIDS Patient Care STDS. 2020;34(4):166–72. doi: 10.1089/apc.2019.0261 32324481

[35] Zanolini A, Sikombe K, Sikazwe I, Eshun-Wilson I, Somwe P, Bolton Moore C, et al. Understanding preferences for HIV care and treatment in Zambia: Evidence from a discrete choice experiment among patients who have been lost to follow-up. PLoS Med. 2018;15(8):e1002636. doi: 10.1371/journal.pmed.1002636 30102693 PMC6089406

[36] Conte M, Eshun-Wilson I, Geng E, Imbert E, Hickey MD, Havlir D, et al. Brief Report: Understanding Preferences for HIV Care Among Patients Experiencing Homelessness or Unstable Housing: A Discrete Choice Experiment. J Acquir Immune Defic Syndr. 2020;85(4):444–9. doi: 10.1097/QAI.0000000000002476 33136742 PMC8028840

